# Shen-yuan-dan capsule inhibits METTL3-mediated m6A methylation to restore autophagy homeostasis and attenuate post-myocardial infarction heart failure

**DOI:** 10.3389/fphar.2026.1661745

**Published:** 2026-03-10

**Authors:** Shuaijie Guo, Siqi Chen, Changxu Xie, Sinai Li, Hongxu Liu, Lei Zhang, Weihong Liu, Mingxue Zhou

**Affiliations:** 1 Beijing Hospital of Traditional Chinese Medicine, Capital Medical University, Beijing, China; 2 Beijing Institute of Chinese Medicine, Beijing, China

**Keywords:** autophagy, heart failure, myocardial infarction, N6-methyladenosine, Traditional Chinese medicine

## Abstract

**Background:**

Heart failure (HF) after myocardial infarction (MI) is a serious health issue. This study investigates the therapeutic effects of Shen-Yuan-Dan Capsule (SYD) on post-MI HF and explores its mechanisms, particularly involving m6A modification and autophagy.

**Methods:**

Network pharmacology and MeRIP-seq were used to predict potential targets. A murine model of post-MI HF was established by ligating the left anterior descending artery in C57BL/6J mice, which were treated with SYD for 6 weeks. Cardiac function, autophagy-related proteins, m6A methylation, and METTL3 levels were assessed. *In vitro*, H9c2 cardiomyocytes were treated with Phenylephrine (PE) and SYD for 24 h, and hypertrophic biomarkers, autophagy proteins, and m6A methylation were measured. METTL3-overexpressing H9c2 cells were also used to investigate SYD’s effects on gene expression.

**Results:**

*In vivo*, SYD treatment significantly improved cardiac function in MI mice, including reduced cardiac hypertrophy, enhanced ejection fraction and fractional shortening, and alleviated myocardial damage, fibrosis, and HF biomarkers. *In vitro*, SYD inhibited PE-induced hypertrophy in H9c2 cells, including a reduction in cell surface area and a decrease in hypertrophic biomarker levels. SYD also inhibited m6A methylation and METTL3 expression. In both MI mice and PE-treated H9c2 cells, SYD lowered m6A levels and METTL3 expression. Bioinformatics analysis identified autophagy-related signaling pathways. Electron microscopy and autophagy marker detection in myocardial tissue and H9c2 cells showed that SYD restored autophagy levels by regulating the mTOR/TFEB autophagy pathway. In METTL3-overexpressing H9c2 cells, SYD treatment reversed the hypertrophy induced by METTL3 overexpression.

**Conclusion:**

SYD alleviates post-MI HF by regulating the mTOR/TFEB autophagy pathway through inhibition of METTL3-mediated m6A modification.

## Graphical Abstract

**Figure:**
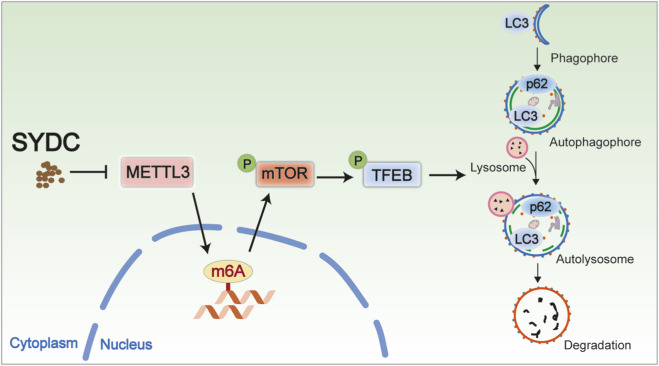

## 1 Introduction

Heart failure (HF), a debilitating syndrome characterized by impaired cardiac output, imposes substantial global health burdens. Myocardial infarction (MI)-induced HF accounts for 10.7%–29.8% of cases within 5 years post-event, largely driven by maladaptive remodeling processes involving cardiomyocyte loss, fibrotic replacement, and progressive ventricular dysfunction (Heidenreich et al., 2022; Gerber et al., 2016; Prabhu and Frangogiannis, 2016). Although contemporary therapies have reduced acute mortality, persistent challenges remain in improving long-term survival and quality of life (Li J. et al., 2015).

Autophagy modulation has emerged as a pivotal therapeutic axis in post-MI HF pathogenesis (Zhang et al., 2022). MI leads to severe cardiac remodeling and the development of HF, during which the levels of autophagy within cardiomyocytes are changed (Kanamori et al., 2011). In addition, autophagy, as a highly conserved mechanism involving lysosome-mediated protein and organelle degradation, serves as a protective mechanism for myocardial cells against ischemic injury damage (Maejima et al., 2013). In this process, transcription factor EB (TFEB) is a pivotal protein that governs lysosomal biogenesis and exocytosis, facilitating the clearance of damaged cells under pathological conditions of MI (Medina et al., 2011). In addition, Mammalian target of rapamycin complex 1 (mTOR), as an upstream regulatory factor of autophagy, phosphorylates specific serine residues in TFEB and primarily influences the subcellular localization of TFEB, contributing to its regulation and acts as a negative regulator of this process (Puertollano et al., 2018; Sciarretta et al., 2014). Murine ischemia models demonstrate that mTOR/TFEB pathway modulation can rescue impaired autophagic flux (Gu et al., 2020). Consequently, modulating autophagic levels through the mTOR/TFEB pathway could emerge as a promising strategy for ameliorating post-MI HF.

Concurrently, epitranscriptomic regulation through N6-methyladenosine (m6A) modification has gained recognition in cardiac pathophysiology. As the predominant internal mRNA modification, m6A governs RNA metabolism through writers like Methyltransferase like 3 (METTL3) (Wu et al., 2020). METTL3 serves as an m6A methyltransferase, catalyzing the transfer of methyl groups onto the N6-adenosine of RNA, thereby modulating RNA methylation levels. Recent study showed that METTL3 also can inhibit TFEB-mediated autophagy in hypoxia/reoxygenation-treated cardiomyocytes (Son et al., 2019). In addition, alterations in m6A levels mediated by METTL3 play a crucial role in cardiac remodeling. Research has also indicated that knockout of METTL3 can promote myocardial cell regeneration in post-MI mice, thereby ameliorating pathological cardiac remodeling (Gong et al., 2021). These findings position METTL3/m6A modulation as a promising therapeutic target.

Given the therapeutic potential of these pathways, Shen-Yuan-Dan Capsules (SYD), a medication approved by the Beijing Municipal Drug Administration and used in clinical practice to treatment in patients with coronary heart disease-induced HF, comprises eight Chinese botanical drugs (*Astragalus mongholicus Bunge [Fabaceae; Astragali radix]*, *Codonopsis pilosula (Franch.) Nannf. [Campanulaceae; Codonopsis radix], Scrophularia ningpoensis Hemsl. [Scrophulariaceae; Scrophulariae radix], Salvia miltiorrhiza Bunge [Lamiaceae; Salviae miltiorrhizae radix et rhizoma], Corydalis yanhusuo (Y.H.Chou and Chun C. Hsu) W.T.Wang ex Z.Y.Su [Papaveraceae; Corydalis rhizoma], Pheretima aspergillum (E. Perrier) [Megascolecidae; Pheretima], Eupolyphaga sinensis Walker [Corydiidae; Eupolyphaga], Hirudo nipponica Whitman [Hirudinidae; Hirudo]*) with identified bioactive compounds including tetrahydropalmatine, harpagoside, salvianic acid A, salvianic acid B, and tanshinone IIA (Xiang et al., 2021; Li et al., 2021). Building upon our prior research findings, it has been demonstrated that SYD can ameliorate HF in zebrafish models by exerting anti-apoptotic and anti-inflammatory effects via activating ROS-induced NF-κB pathway (Li et al., 2021). In addition, our previous studies suggested that SYD inhibited foam cell formation by promoting autophagy via the inhibition of the PI3K/AKT/mTORC1 signaling pathway (Zhou et al., 2019). However, the mechanisms of SYD in the context of MI-induced HF remain unclear. This study integrates *in vivo* and *in vitro* approaches to investigate whether SYD ameliorates post-MI HF by regulating METTL3/m6A-dependent mTOR/TFEB-autophagy signaling.

## 2 Materials and methods

### 2.1 Drugs and reagents

SYD was provided by Beijing TCM Hospital (Beijing, China, Z20053327), and thestock liquor of 20 mg/mL was prepared with 0.5% carboxymethyl cellulose sodium (CMC-Na) before the experiment. SYD consists of eight botanical drugs: *Astragalus mongholicus* Bunge [Fabaceae; Astragali radix] (60 g), *Codonopsis pilosula* (Franch.) Nannf. [Campanulaceae; Codonopsis radix] (60 g), *Scrophularia ningpoensis* Hemsl. [Scrophulariaceae; Scrophulariae radix] (60 g), *Salvia miltiorrhiza* Bunge [Lamiaceae; Salviae miltiorrhizae radix et rhizoma] (60 g), *Corydalis yanhusuo* (Y.H.Chou and Chun C. Hsu) W.T.Wang ex Z.Y.Su [Papaveraceae; Corydalis rhizoma] (40 g), *Pheretima aspergillum* (E. Perrier) [Megascolecidae; Pheretima] (40 g), *Eupolyphaga sinensis* Walker [Corydiidae; Eupolyphaga] (24 g), and *Hirudo nipponica* Whitman [Hirudinidae; Hirudo] (24 g). All botanical drugs were obtained from the Pharmacy of Beijing Hospital of Traditional Chinese Medicine (Beijing, China) and comply with the standards of the *Pharmacopoeia of the People’s Republic of China* (2020 Edition). The materials were authenticated by Professor Jiankun Wu (Pharmacy Department of Beijing TCM Hospital). Voucher specimens (No. Z20233327]) have been deposited in the Beijing Institute of Chinese Medicine.

To ensure chemical consistency, the SYD extract was characterized using ultra-high-performance liquid chromatography-tandem mass spectrometry (UPLC–MS/MS) on a Waters system with a HESI-II probe, Separation was performed on an Acquity UPLC HSST3 column (1.8 μm × 2.1 mm × 100 mm) at 45 °C, using a mobile phase of 0.1% formic acid in water (A) and 0.1% formic acid in methanol (B) with a flow rate of 0.3 mL/min. The HESI-II spray voltages were 3.7 kV (positive) and 3.5 kV (negative), with a vaporizer temperature of 320 °C. Data acquisition was processed via Waters Masslynx 4.1. The representative total ion chromatograms (TIC) and identified metabolites are provided in Supplementary Figure S1.

The botanical drugs were mixed and soaked in distilled water for 1 h, followed by boiling for 1 h and filtration. The residue was decocted again with distilled water for 30 min and filtered. The two extracts were combined, concentrated to 100 mL, and clarified. The supernatant was collected, pre-frozen at −80 °C, and freeze-dried using a vacuum freeze-dryer. The total weight of the starting botanical drugs was 368 g. After the extraction and lyophilization process, 68 g of freeze-dried powder was obtained. The final drug-extract ratio (DER) was 5.41:1, corresponding to a yield of 18.48% (w/w). The freeze-dried powder was stored in a desiccator at room temperature. Sacubitril/valsartan sodium tablet (SVST) (Lot #H20170363), was purchased from Novartis Singapore Pharmaceutical Manufacturing Private. Ltd. (Beijing, China).

Isoflurane (R510) was purchased from RWD Life Science Co., Ltd. (Shenzhen, China). PE (S2569) was purchased from Slleck (Houston, United States). Dulbecco’s modified Eagle’s medium (DMEM) (SH30243.01) was purchased from Hyclone (Utah, United States). Fetal bovine serum (10091-148), penicillin-streptomycin (15140122), 0.25% Trypsin-EDTA (25200056), BCA Protein Assay Kit (23225) and TRIzol (15596026CN) were purchased from Thermo fisher (Massachusetts, United States). The ELISA-kit of BNP(LV20905), β-MHC (LV20784), TGF-β1 (LV20903), ANP (LV20904) and NTpro-BNP (LV30938) were purchased from Animal union Biotechnology Co., Ltd (Shanghai, China). Cell Counting Kit-8 (CK04) was purchased from Dojindo North. (Beijing, China). Paraformaldehyde, 4% (P1110), Hematoxylin-Eosin (H&E) Stain Kit (G1120), Modified Masson’s Trichrome Stain Kit (G1346), Triton X100 (IT9100), RIPA buffer (R0010), Protein Phosphatase Inhibitor (P1206), Methylene Blue Solution, 0.2% (G1301), Glutaraldehyde (P1127) were purchased from Solarbio (Beijing, China). Alpha Actin Polyclonal antibody (23660-1-AP), CoraLite488-conjugated Goat Anti-Rabbit IgG (H + L) (SA00013-2), m6A Monoclonal antibody (68055-1-Ig), Goat serum (B900780), Prestained Protein marker (PL00001), Extra Range Prestained Protein Marker (PL00003), Beta Tubulin Polyclonal antibody (10094-1-AP), HRP-conjugated Affinipure Goat Anti-Mouse IgG (H + L) (SA00001-1), HRP-conjugated Affinipure Goat Anti-Rabbit IgG (H + L) (SA00001-2) were purchased from Proteintech (Wuhan, China). LC3A/B Rabbit mAb (12741), SQSTM1/p62 Rabbit mAb (23214), Beclin-1 Rabbit mAb (3495), Phospho-mtor (Ser2448)Rabbit mAb (5536), Phospho-TFEB (Ser211) (E9S8N) Rabbit mAb (37681)TFEB Rabbit mAb (32361), METTL3 Rabbit mAb (96391), GAPDH Rabbit mAb (97166) were purchased from Cell Signaling Technology (Massachusetts, United States).

### 2.2 Animals

Six-week-old male C57BL/6 mice (weighing 21–23 g) were purchased from Beijing Vital River Laboratory Animal Technology Co., Ltd. (Beijing, China). During the feeding period, mice were kept under a 12-h alternating light cycle (8:00-20:00), with room temperature maintained at approximately 23 °C and humidity kept between 30% and 40%. Free access to food and water was provided throughout the experiment. All mice were reared in the animal room for 2 weeks and then used for subsequent animal experiments. All the animal experiments in this study were approved by the Animal Ethics Committee of the Beijing Institute of Traditional Chinese Medicine (No. BJTCM-M-2024-09-04).

### 2.3 Animal modelling, grouping and pharmacological treatments

The left anterior descending coronary artery (LAD) ligation was performed to establish the MI-induced HF model following the methods in our previous study (Li et al., 2023). Briefly, anesthetized mice were attached to an animal ventilator, a left thoracotomy was conducted, and the LAD was occluded by ligating the left ventricle 1–1.5 mm from the left atrial appendage with 6/0 silk yarn. Mice in the Sham group were subjected to the same thoracotomy without ligating the LAD. During the operation, the white of left ventricular anterior wall can be used as an important indicator to evaluate the success of the operation. Finally, the ST segment elevation greater than 0.2 mV indicates the success of myocardial infarction model.

The mice were randomly allocated into six groups: sham, model, low-dose SYD (SYD-L), medium-dose SYD (SYD-M), high-dose SYD (SYD-H), and positive control (SVST).

After surgery, the mice on the SYD-L group, SYD-M group, and SYD-H group were treated with 0.3, 0.6, and 1.2 g/kg/d of SYD by gavage, respectively. The dosage of SYD-M is converted according to the 9.1 conversion factor according to the clinical recommend dose (4.2 g/day) of adult daily medication (Li et al., 2022). The mice on the SVST group were treated 60 mg/kg/d of SVST (Liu et al., 2021). The Sham and Model groups were administered the equal amounts of 5% CMC-Na. All mice received corresponding drug intervention for 6 weeks.

### 2.4 Cells

Embryonic rat cardiomyocyte cell line (H9c2 cells, obtained from the Cell Resource Center of the Institute of Basic Medical Sciences, Chinese Academy of Medical Sciences, China) were maintained at 37 °C in a humidified atmosphere containing 5% CO2. The culture medium consisted of DMEM, 10% fetal bovine serum, 100 μg/mL penicillin, and 100 μg/mL streptomycin. Lentiviral vectors were constructed by Beijing XBHCbio Co (Beijing, China) and used to overexpress METTL3 in H9c2 cells. The lentivirus for overexpressing METTL3 (OE-METTL3) and the negative control lentiviral vector were prepared and titrated to 5 × 10^8^ TU/mL.

### 2.5 Cells modelling, grouping and treatments

The H9c2 cell hypertrophy model was constructed by inducing PE (50 μM) for 24 h (Chen et al., 2024). The cell hypertrophy model evaluates the success of the model by increasing the cell surface area.

Weigh 0.1 g of SYD freeze-dried powder and dissolve it in 10 mL of DMEM. Vortex thoroughly and filter the solution using a 0.22 μm filter to obtain a sterile stock solution of SYD freeze-dried powder. Prior to the experiment, dilute the stock solution to the desired concentration.

The experimental cell treatments were as follows: Control group (no intervention), Model group (induced with 50 μM PE for 24 h), SYD-L group (treated with 0.5 mg/mL SYD with 50 μM PE for 24 h), SYD-M group (treated with 1 mg/mL SYD with 50 μM PE for 24 h) and SYD-H group (treated with 2 mg/mL SYD with 50 μM PE for 24 h).

Blank group, Negative vector control group (VE-C) and Overexpression-METTL3 control group (OE-C) treated with no intervention; Negative vector model group (VE-M) and Overexpression-METTL3 model group (OE-M) treated with 50 μM PE for 24 h; Negative vector SYD group (VE-S) and Overexpression-METTL3 SYD group (OE-S) treated with 1 mg/mL SYD with 50 μM PE for 24 h.

### 2.6 Echocardiographic analysis

On 6 weeks after surgery, the mice were anesthetized with 1.5% isoflurane and echocardiography was conducted with a Vevo 3100 Imaging System (Visual Sonics Inc, Canada). Briefly, an MI-mode echocardiogram was used to analyze cardiac function indicators, including left ventricular ejection fraction (EF), left ventricular fractional shortening (FS), left ventricular internal dimension at end-diastole (LVIDd), and left ventricular internal dimension at end-systole (LVIDs).

### 2.7 Histological examination

On 6 weeks after surgery, mouse hearts were removed and fixed in 4% paraformaldehyde for 24 h. The hearts were then dehydrated, embedded in paraffin, and sectioned for 5 μm. Masson’s trichrome staining was used to examine cardiac fibrosis. H&E staining was used to explore basic pathological changes. After staining, sections were observed and photographed under a digital pathology slide scanner (Aperio CS2, Leica, Germany).

### 2.8 ELISA

The mouse serum or H9c2 cardiomyocyte culture supernatants were collected and used to measure protein levels of specific biomarkers using ELISA kits according to the manufacturer’s instructions. Briefly, samples and standards were added to 96-well plates pre-coated with capture antibodies. The plates were incubated at room temperature for 1–2 h, followed by washing to remove unbound components. Next, detection antibodies were added and incubated, followed by the addition of enzyme-linked secondary antibodies. After thorough washing, a substrate solution was applied to develop the color reaction. The reaction was stopped using a stop solution, and absorbance was measured at 450 nm using a microplate reader. The concentration of the target protein was calculated based on the standard curve.

### 2.9 Transmission electron microscope (TEM)

Heart tissues or H9c2 cells were removed and immediately washed with PBS. After fixation with electron microscope solution for 3 h, fixation with PBS containing 1% osmic acid for 2 h, dehydration was carried out in gradient ethanol. Finally, it was embedded and sliced by an embedding agent, uranium-lead double dyeing, and observed by TEM.

### 2.10 Western blotting

Heart tissues or H9c2 cells were homogenized in radio-immunoprecipitation assay lysis buffer, and total protein concentrations were determined by using a bicinchoninic acid protein assay kit. Equal amounts of protein extracts were separated using 10% sodium dodecyl sulfate-polyacrylamide gel electrophoresis. Proteins were then transferred to methanol-preactivated polyvinylidene difluoride membranes. Membranes were then blocked with 5% skim milk for 1 h at room temperature and incubated with primary antibodies overnight at 4 °C. After washing with TBS-T, membranes were incubated with a horseradish peroxidase-conjugated secondary antibody for 1 h at room temperature. Finally, proteins were visualized using an enhanced chemical luminescent system (Pierce Biotechnology, Inc., Rockford, United States), and bands were imaged using a chemiluminescence imaging system.

### 2.11 Dot-blotting

After the total RNA was extracted from the mice heart tissue or H9c2 cells, 2ul of total RNA was added to the NC membrane in a unified concentration. Crosslinking 1 h under 254 nm UV lamp. Overnight incubation using 0.1% m6A antibody following closure with TBST containing 5% skim milk powder. The next day, imaging was performed using chemiluminescence.

### 2.12 Cell viability

Cell viability was detected by using the CCK-8 assay kit. Briefly, H9c2 cells were seeded at a density of 1 × 10^6^ cells per well in a 96-well plate and incubated overnight to allow for adherence. Subsequently, H9c2 cells were treated with varying concentrations of PE (0, 25, 50, 100, and 200 μmol/L) and SYD (0, 1, 2, 3, 4, 5, and 6 mg/mL) for 24 h. After treatment, CCK-8 solution was added, and the H9c2 cells were incubated at 37 °C for 1 h. The absorbance at 450 nm was measured using a microplate reader, and cell viability was calculated accordingly.

### 2.13 Immunofluorescence

Cells were fixed, permeabilized, and blocked, followed by incubation with an α-actin antibody overnight at 4 °C. The next day, cells were incubated with a fluorescently labeled secondary antibody for 1 h. A mounting medium containing DAPI was added to prevent fluorescence quenching. The cells were then examined using an inverted immunofluorescence microscope.

### 2.14 Real-time quantitative PCR

Total RNA was extracted from experimental cells using TRIzol reagent. The extracted RNA was then reverse transcribed into cDNA using the HiScript III 1st Strand cDNA Synthesis Kit (+gDNA wiper). The mRNA levels of METTL3, mTOR, and TFEB were quantified using the SYBR Green I incorporation method. The reaction program was set as follows: Initial Denaturation: 95 °C for 10 min. Amplification Cycles (45 cycles): Denaturation: 95 °C for 15 s. Annealing: 60 °C for 60 s. Melting Curve Analysis: 95 °C for 15 s. Cooling to 60 °C for 1 min. Heating to 95 °C for 30 s. Fluorescence readings were recorded. GAPDH was used as the internal control. The relative mRNA expression levels were determined using the 2^−ΔΔCT^ method. Primer sequences are listed in Table 1.

**TABLE 1:** Primers for RT-qPCR.

Gene	Primer sequence (5′to 3′)	Gene	Primer sequence (5′to 3′)
METTL3	ATG​ACG​CAC​ATC​CCA​CTC​T	mTOR	ACA​AGA​ATG​GTG​CCG​AAA​G
	GCTCTGCACGCCGTTTCT		TGGCTGGTTGGGGTCA
TFEB	TGCCCTGCCGACCTGACT	GAPDH	CGTATCGGACGCCTGGTT
	CTT​TCT​TCT​GCC​GTT​CCT​T		AGG​TCA​ATG​AAG​GGG​TCG​TT

### 2.15 m6A MeRIP-Seq

The m6A MeRIP-Seq service was provided by CloudSeq Inc. (Shanghai, China). Total RNA was subjected to immunoprecipitation with the GenSeq® m6A MeRIP Kit (GenSeq Inc.) by following the manufacturer’s instructions. Briefly, RNA was randomly fragmented to ∼200 nt by RNA Fragmentation Reagents. Protein A/G beads were coupled to the m6A antibody by rotating at room temperature for 1 h. The RNA fragments were incubated with the bead-linked antibodies and rotated at 4 °C for 4 h. After incubation, the RNA/antibody complexes were washed for several times, and then, captured RNA was eluted from the complexes and purified. RNA libraries for IP and input samples were then constructed with GenSeq® Low Input Whole RNA Library Prep Kit (GenSeq, Inc.) by following the manufacturer’s instructions. Libraries were qualified using Agilent 2100 bioanalyzer (Agilent) and then sequenced.

Raw reads (Raw Data) are generated after sequencing on a sequencer, image analysis, base identification and QC. Q30 was first used for quality control. Then, cutadapt software (v1.9.3) was used to remove splice information and remove low quality reads to obtain high quality clean reads. The clean reads were matched to the reference genome using Hisat2 (v2.0.4). Methylated genes in each sample were then identified using MACS (1.4.2). Differential methylation gene identification was performed using diffReps (1.55.6). The peaks located mRNA were screened using our own programs and annotated accordingly.

### 2.16 Network pharmacology analysis

Drug ingredients were obtained from TCMSP database and previous literature. Search for drug ingredient targets through HERB and SwissTargetPrediction databases. Relevant HF targets were collected through GeneCard, DISGENET and TTD databases. Finally, the “Botanical drug-Ingredient-Target network” was constructed by cytoscape 3.7.0. The core targets were imported into David database for KEGG and GO enrichment analysis.

### 2.17 Statistical analysis

Quantitative data are expressed as mean ± SEM. Data were statistically analyzed using Graphad Prism 9.5 software, and Ordinary one-way ANOVA was used for three or more groups, and then Tukey’s multiple comparison test was conducted. P<0.05 was considered statistically significant.

## 3 Results

### 3.1 SYD attenuates cardiac remodeling and restores cardiac function in MI mice

**FIGURE 1:**
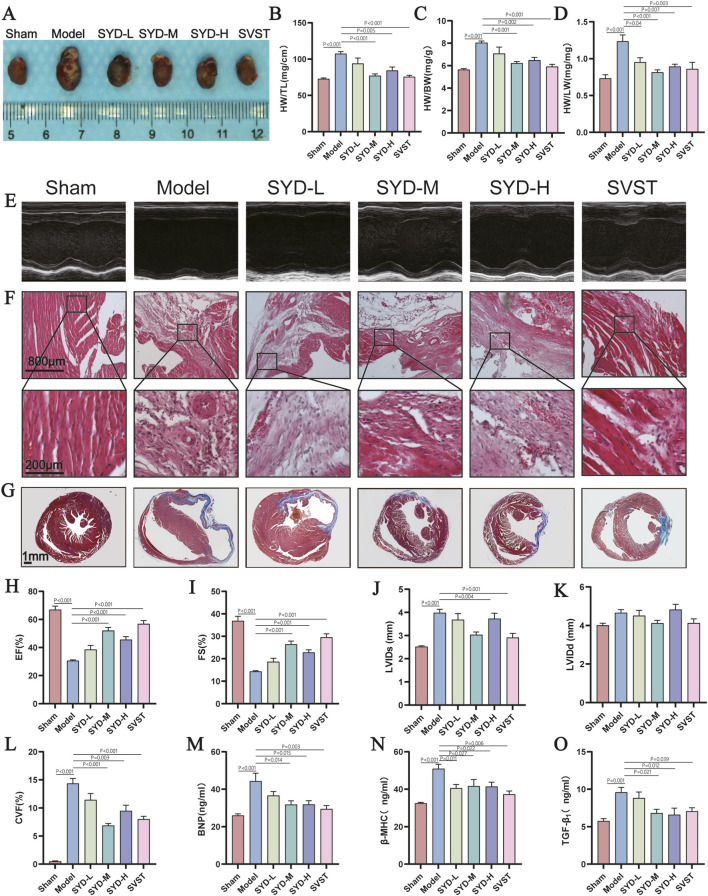
SYD inhibited myocardial remodeling and improved cardiac function in mice with post-MI HF. **(A)** Representative images of cardiac morphology for mice in each group at 6 weeks post-surgery. **(B–D)** Levels of HW/BW, HW/LW, and HW/TL ratios in mice from each group. (n = 10). **(E)** Typical echocardiographic images of mice from each group at 6 weeks post-surgery. **(F,G)** Representative micrographs of HE and Masson staining in different groups. **(H–K)** Statistical graphs of EF, FS, LVID, and LVIDd for mice in each group. (n = 10). **(L)** Collagen volume fraction. **(M–O)** Levels of BNP, β-MHC, TGF-β1 in the serum of mice from different groups. (n = 6). All column diagram data are shown as mean ± SEM. SYD-L: Low-dose SYD group; SYD-M: Medium-dose SYD group; SYD-H: High-dose SYD group; Sacubitril/valsartan sodium tablet positive control (SVST).

*In vivo*, we established a murine MI model through permanent left anterior descending (LAD) coronary artery ligation. Six-week pharmacological intervention revealed cardioprotective effects of SYD. Initial evaluation of cardiac remodeling indices demonstrated that MI mice exhibited significantly elevated heart weight-to-body weight (HW/BW), heart weight-to-lung weight (HW/LW), and heart weight-to-tibia length (HW/TL) ratios compared to sham controls (Figures 1A–D), indicative of pathological cardiac enlargement. These ratios were substantially normalized by medium/high-dose SYD and SVST treatment. Echocardiographic assessment revealed deteriorated systolic function in MI mice (Figure 1E), characterized by reduced ejection fraction (EF) and fractional shortening (FS), along with increased left ventricular internal diameter during systole (LVIDs). Remarkably, SYD medium/high doses and SVST significantly ameliorated these functional impairments (Figures 1H–K). Histopathological analysis via hematoxylin-eosin (HE) and Masson’s trichrome staining demonstrated extensive myocardial necrosis, interstitial edema, inflammatory infiltration, and collagen deposition in MI mice, which were mitigated by SYD and SVST treatments (Figures 1F,G). ELISA quantification of serum biomarkers associated with HF and remodeling including B-type natriuretic peptide (BNP), β-myosin heavy chain (β-MHC), and transforming growth factor-β1 (TGF-β1) revealed significant reductions in SYD-M, SYD-H, and SVST groups compared to MI controls (Figures 1L–O). Collectively, these findings substantiate SYD’s therapeutic potential in ameliorating post-MI cardiac remodeling and functional deterioration.

### 3.2 SYD promotes autophagy and inhibits m6A methylation in MI mice

**FIGURE 2:**
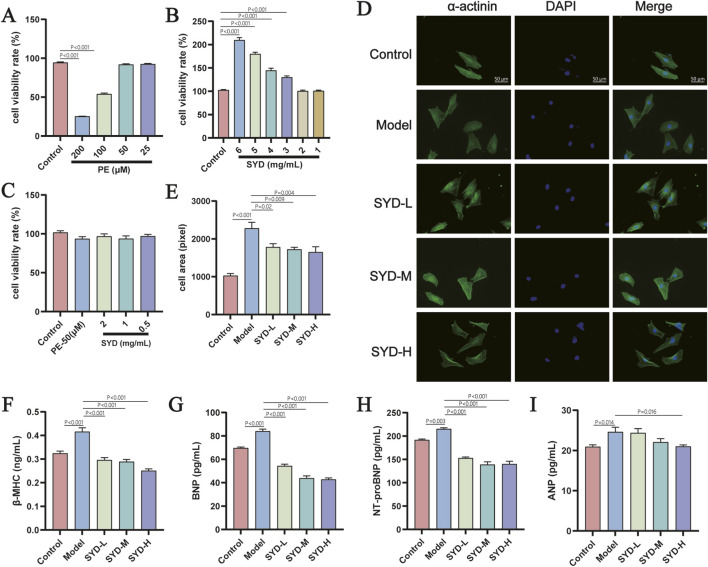
SYD inhibits PE-induced hypertrophy of H9c2 cardiomyocytes. **(A)** Cell viability rate of H9c2 cells under gradient metric PE intervention (n = 6). **(B)** Cell viability rate of H9c2 cells under gradient metric SYD intervention (n = 6). **(C)** Cell viability rate of H9c2 cells under gradient metric PE and SYD intervention (n = 6). **(D)** Immunofluorescence staining of α-actinin. **(E)** cell area of each group (n = 4), Scale bars, 50 μm. **(F–I)** The content of hypertrophy markers (β-MHC, BNP, NT-proBNP, ANP) in the supernatant of H9c2 cells in each group (n = 8). All column diagram data are shown as mean ± SEM. SYD-L: Low-dose SYD group; SYD-M: Medium-dose SYD group; SYD-H: High-dose SYD group.

After confirming the anti-HF effects of SYD in MI mice, we screened the drug concentrations of PE (50 μM) and SYD (0.5/1/2 mg/mL) through CCK-8 assays to determine the intervention doses, which were used to induce hypertrophy in H9c2 cells and anti-hypertrophy (Figures 2A–C). Immunofluorescence staining of α-actin in H9c2 cells was performed to measure the cell surface area, which showed a significant reduction in cell size after SYD intervention (Figures 2D,E). ELISA quantification of hypertrophic biomarkers (β-MHC, BNP, NT-proBNP, ANP) in the culture supernatant indicated that SYD treatment decreased the levels of these markers (Figures 2F–I). Collectively, these results demonstrate that SYD can suppress PE-induced cardiomyocyte hypertrophy.

### 3.3 SYD modulates m6A methylation via METTL3 regulation

**FIGURE 3:**
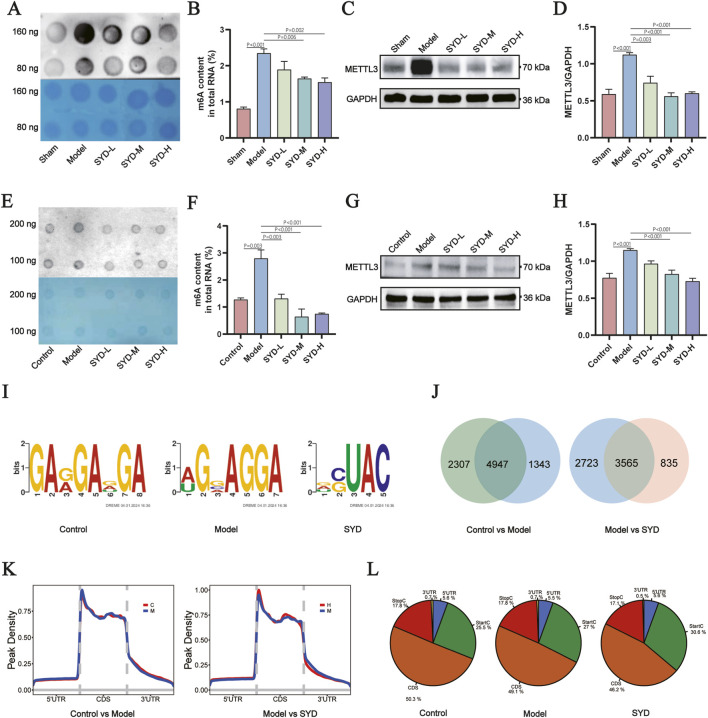
The effect of SYD on m6A methylation both *in vitro* and *in vivo*. **(A,B)** Representative dot-blot images and m6A methylation levels of total RNA in the hearts of mice in each group (n = 3). **(C,D)** Representative WB images of whole-cell cardiac homogenates for METTL3. Quantitative analysis of METTL3 normalized to GAPDH (n = 3). **(E)** Representative dot-blot images and m6A methylation levels of total RNA in the H9c2 in each group. **(F)** Quantitative analysis of m6A normalized to total RNA (n = 5). **(G)** Representative WB images of H9c2 for METTL3. **(H)** Quantitative analysis of METTL3 normalized to GAPDH (n = 5). **(I)** The analysis of motif. **(J)** Veen plot of peak counts between control group, model group, and SYD group. **(K)** Methylation density in the metagene region. The X-axis represents positional information, while the Y-axis represents peak density. **(L)** Coordinate information of each peak group and corresponding reference genome annotation files. All column diagram data are shown as mean ± SEM. SYD-L: Low-dose SYD group; SYD-M: Medium-dose SYD group; SYD-H: High-dose SYD group.

Having clarified the therapeutic effects of SYD on post-MI HF mice and hypertrophic cardiomyocytes, we proceeded to investigate its underlying mechanisms. Dot blot and immunoblot analyses demonstrated elevated myocardial m6A levels and METTL3 expression in post-MI HF mice, which were reversed by SYD treatment (Figures 3A–D). Consistently, SYD normalized PE-induced m6A hypermethylation and METTL3 upregulation in H9c2 cells (Figures 3E–H). Methylated RNA immunoprecipitation sequencing (MeRIP-seq) identified differential m6A peak distribution among control, PE-induced, and SYD-treated groups. Motif analysis confirmed conserved RRACH sequences (R = purine, A = m6A, H = non-guanine) across groups with intergroup variations (Figure 3I). Comparative analysis revealed 7,254 vs. 6,290 m6A peaks between control and model groups (4,947 shared), and 6,288 vs. 4,400 peaks between model and SYD groups (3,565 shared) (Figure 3J). Metaplot analysis showed distinct 5′UTR methylation patterns across groups (Figure 3K), with peak distribution differences in coding sequences (CDS) and start/stop codon regions (Figure 3L). These findings establish SYD-mediated regulation of differential m6A methylation landscapes.

### 3.4 Bioinformatics analysis SYD’s therapeutic targets and pathways

**FIGURE 4:**
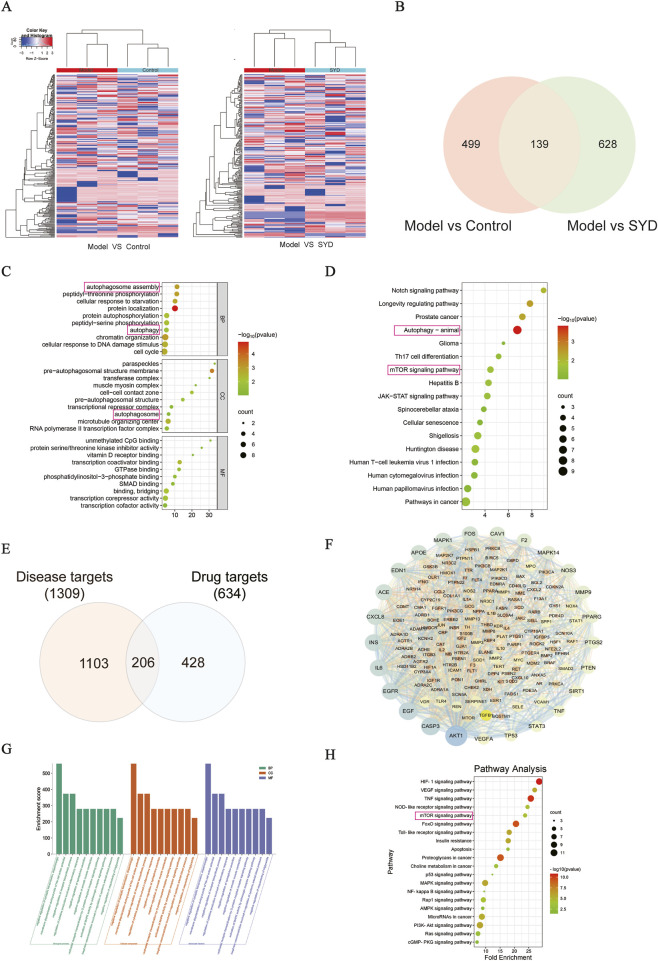
Differential methylation analysis and network pharmacology prediction of SYD in the treatment of post-MI HF. **(A)** Heatmap of differential methylated mRNAs. **(B)** Venn diagram of differential methylated mRNAs between the two groups. **(C,D)** GO enrichment analysis and KEGG of 139 modified genes. **(E)** Venn digram of disease and drug targets. **(F)** Target protein interaction network diagram. **(G,H)** Enrichment analysis diagram of GO and KEGG.

To systematically explore the pharmacological basis of SYD in treating post-MI HF and identify potential targets regulated by m6A modification, we performed an integrative analysis of MeRIP-seq data and network pharmacology. Differential m6A methylation analysis identified 638 DMGs between the Model and Control groups, and 767 DMGs between the Model and SYD groups (Figure 4A), with 139 overlapping genes identified as potential key targets mediating the cardioprotective effects of SYD (Figure 4B). Functional enrichment analyses (GO and KEGG) for these 139 genes significantly highlighted biological processes related to autophagosome assembly, autophagy regulation, and the mTOR signaling pathway (Figures 4C,D). Concurrently, network pharmacology was employed to provide a global profile of SYD’s multi-target landscape. A total of 186 potential bioactive metabolites from SYD were identified (Table 2), yielding 634 component targets, which intersected with 1,309 disease-related targets to produce 206 potential therapeutic targets (Figure 4E). The “drug-metabolite-target” network and topological analysis further positioned central autophagy regulators, such as mTOR and SQSTM1 (p62), as core nodes (Figures 4F,G). Importantly, enrichment analyses of these network targets independently confirmed the prominence of cardiac contractility regulation and the mTOR/autophagy axis (Figure 4H), showing high consistency with the MeRIP-seq findings. Collectively, these dual bioinformatic perspectives converge on the regulation of autophagic homeostasis through the mTOR pathway, providing a robust rationale for the subsequent mechanistic validation of the METTL3/m6A -mTOR/TFEB axis.

**TABLE 2:** Potential metabolites of SYD.

Botanical drug name	Chemical components	Botanical drug name	Chemical components	Botanical drug name	Chemical components
*Salviae Miltiorrhizae Radix et Rhizoma*	Baicalin	*Codonopsis Radi*	poriferasta-7,22E-dien-3beta-ol	*Corydalis Rhizoma*	bicuculline
	cryptotanshinone		Perlolyrine		Bifendate
	Danshenol A		Diop		Calycosin
	Danshenol B		Stigmasterol		Capaurine
	danshenspiroketallactone		7-Methoxy-2-methyl isoflavone		Cavidine
	dan-shexinkum d		Spinasterol		Clarkeanidine
	Dehydrotanshinone II A		Chrysanthemaxanthin		coptisine
	deoxyneocryptotanshinone		Frutinone A		Corydaline
	digallate		luteolin		Corydalmine
	dihydrotanshinlactone		Taraxerol		Corydine
	Dihydrotanshinone I		stigmast-7-enol		Corynoloxine
	epidanshenspiroketallactone		3-beta-Hydroxymethyllenetanshiquinone		Cryptopin
	formyltanshinone		methyl icosa-11,14-dienoate		dehydrocavidine
	isocryptotanshi-none		5alpha-Stigmastan-3,6-dione		Dehydrocorybulbine
	isoimperatorin		7-(beta-Xylosyl)cephalomannine_qt		dehydrocorydaline
	Isotanshinone II		Daturilin		Dehydrocorydalmine
	manool		glycitein		demethylcorydalmatine
	Methylenetanshinquinone		Spinoside A		Dihydrochelerythrine
	Miltionone I		11-Hydroxyrankinidine		Dihydrosanguinarine
	miltionone II	*Astragali Radix*	norglaucing		FA
	miltipolone		palmatine		formononetin
	Miltirone		pontevedrine		Fumarine
	miltirone II		pseudocoptisine		hederagenin
	neocryptotanshinone		quercetin		Hyndarin
	neocryptotanshinone ii		sanguinarine		isocorybulbine
	poriferast-5-en-3beta-ol		saulatine		Isocorypalmine
	Poriferasterol		sitosterol		isorhamnetin
	prolithospermic acid		Stigmasterol		Izoteolin
	przewalskin a		stylopine		Jaranol
	przewalskin b		Tetrahydrocorysamine		kaempferol
	Przewaquinone B		tetrahydroprotopapaverine		leonticine
	przewaquinone c	*Scrophulariae Radix*	sugiol		Mairin
	Przewaquinone E		scropolioside D		N-methyllaurotetanine
	przewaquinone f		sitosterol		tetrahydropalmatine
	salvianolic acid A		beta-sitosterol	Pheretima	3-Benzyl hypoxanthine
	salvianolic acid G		scropolioside A_qt		4-Aminobutanoic acid
​	Salvilenone		14-deoxy-12(R)-sulfoandrographolide		Abieta-8,11,13-trien-18-ol
	salvilenone I		paeoniflorin_qt		Adenine
	salviolone		harpagoside_qt		Arachidonic acid
	sclareol	Hirudo	11-Hexadecenoic acid methyl ester		Cycloleucine
	tanshinaldehyde		adenine		Dihydrocapsaicin
	Tanshindiol B		Chimyl alcohol		DL-Tryptophan
	tanshinone VI		DL - tryptophan		D-tert-Leucine
	tanshinone II A		DL-Isoleucine		Eicosapentaenoic Acid
	α-amyrin		DL-Leucine		Guanine
Eupolyphaga sinensis	Acetophenone		DL-Lysine		H-Leu-Phe-OH
	DL-Tryptophan		DL-Methionine		L-(-)-Tyrosine
	D-tert-Leucine		DL-Phenylalanine		Lauric acid-13C
	Genkwanin		DL-Tyrosine		L-glutamic acid
	Heptanedione		DL-Valine		Linolelaidic acid
	Hexadecadienoic acid		hexanal		L-Lysine
	L-(-)-Tyrosine		hirudinoidine A		L-Methionine
	Lauric acid		histamine		Myristic acid
	L-glutamic acid		hypoxanthine		Myristoleic acid
	L-Histidine		indole- 3 - carboxaldehyde		Nicotinic acid
	l-isoleucine		L- glutamic acid		Palmitoleic acid
	L-Lysine		methyl 12 - methyltetradecanoate		Pentadecanoic acid
	L-Methionine		methyl 4 -methy tetradecanoate		phenylalanine
	Myristic acid		nicotimic acid		Tridecylic acid
	Myristic acid		palmitic acid		Valine
	Phenol		Succinic Acid		
	phenylalanine		xanthine		
	Valine				
	Vitamin A				

### 3.5 SYD enhances autophagy in Post-MI hearts

**FIGURE 5:**
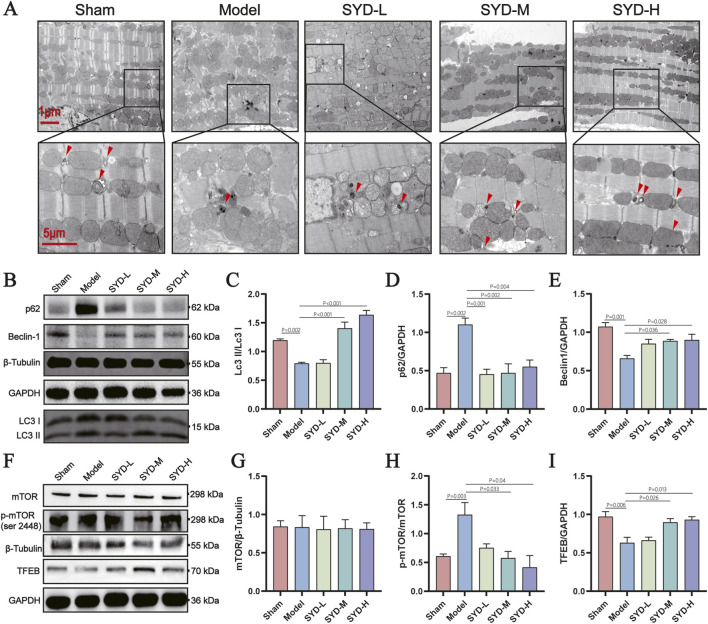
SYD promotes autophagy in mice with HF after MI. **(A)** Representative TEM photographs of the peri-infarct area of the mice heart (The red arrow indicates the lysosome). **(B)** Representative WB images of whole-cell cardiac homogenates for LC3, Beclin-1, p62 and house-keeping genes. **(C)** Quantitative analysis of LC3 normalized to β-Tubulin (n = 3). **(D,E)** Quantitative analysis of p62 and Beclin-1 normalized to GAPDH (n = 3). **(F)** Representative WB images of whole-cell cardiac homogenates for mTOR, p-mTOR, TFEB and house-keeping genes. **(G–I)** Quantitative analysis of mTOR normalized to β-Tubulin, p-mTOR normalized to mTOR and TFEB normalized to GAPDH (n = 3). All column diagram data are shown as mean ± SEM. SYD-L: Low-dose SYD group; SYD-M: Medium-dose SYD group; SYD-H: High-dose SYD group; Sacubitril/valsartan sodium tablet positive control (SVST).

Ultrastructural analysis via transmission electron microscopy (TEM) revealed SYD-mediated mitigation of myocardial hypertrophy and organelle swelling, accompanied by increased autolysosomes (Figure 5A). Immunoblotting confirmed SYD dose-dependently upregulated autophagy markers (LC3-II/I ratio, Beclin-1) while reducing p62 accumulation (Figures 5B–E). Concurrently, SYD suppressed mTOR phosphorylation and enhanced TFEB expression (Figures 5F–I), indicating activation of autophagy-lysosomal pathways.

### 3.6 SYD inhibits autophagy in PE-Induced hypertrophic H9c2 cardiomyocytes

**FIGURE 6:**
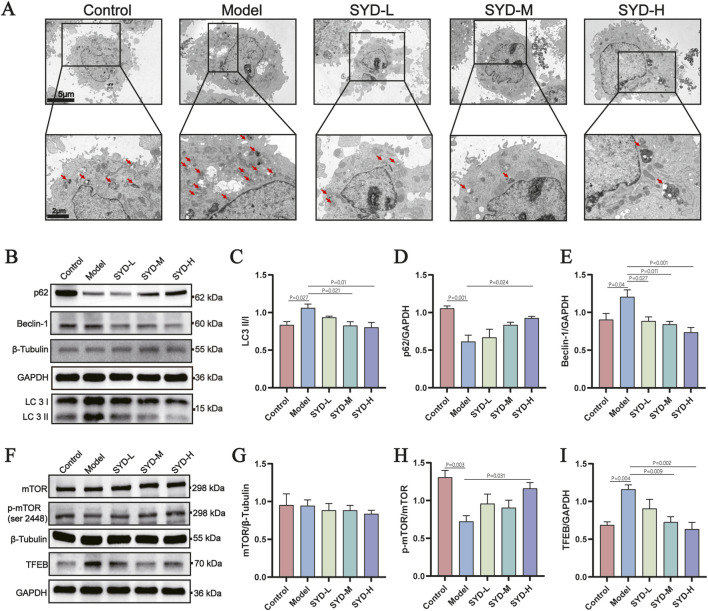
SYD inhibits autophagy in PE-induced hypertrophic H9c2 cardiomyocytes. **(A)** Representative TEM photographs of the peri-infarct area of the H9c2 cell. **(B)** Representative WB images of H9c2 cell for LC3, Beclin-1, p62 and house-keeping genes. **(C)** Quantitative analysis of LC3 normalized to β-Tubulin (n = 5). **(D,E)** Quantitative analysis of p62 and Beclin-1 normalized to GAPDH (n = 5). **(F)** Representative WB images of whole-cell cardiac homogenates for mTOR, p-mTOR, TFEB and house-keeping genes. **(G–I)** Quantitative analysis of mTOR normalized to β-Tubulin, p-mTOR normalized to mTOR and TFEB normalized to GAPDH (n = 5). All column diagram data are shown as mean ± SEM. SYD-L: Low-dose SYD group; SYD-M: Medium-dose SYD group; SYD-H: High-dose SYD group.

Contrasting with *in vivo* findings, PE-induced autophagic hyperactivity in H9c2 cells was counteracted by SYD treatment, as evidenced by TEM (Figure 6A) and immunoblot analysis of autophagy markers (Figures 6B–E). Subsequently, we examined the expression levels of the mTOR/TFEB pathway and found that SYD intervention enhanced mTOR phosphorylation and reduced TFEB protein expression in hypertrophic H9c2 cells (Figures 6F–I). Interestingly, the autophagy levels exhibited opposing trends *in vivo* and *in vitro*, which we believe reflects disrupted autophagic homeostasis and highlights the bidirectional nature of autophagy.

### 3.7 SYD regulates the mTOR/TFEB autophagy pathway through METTL3-mediated m6A modification

**FIGURE 7:**
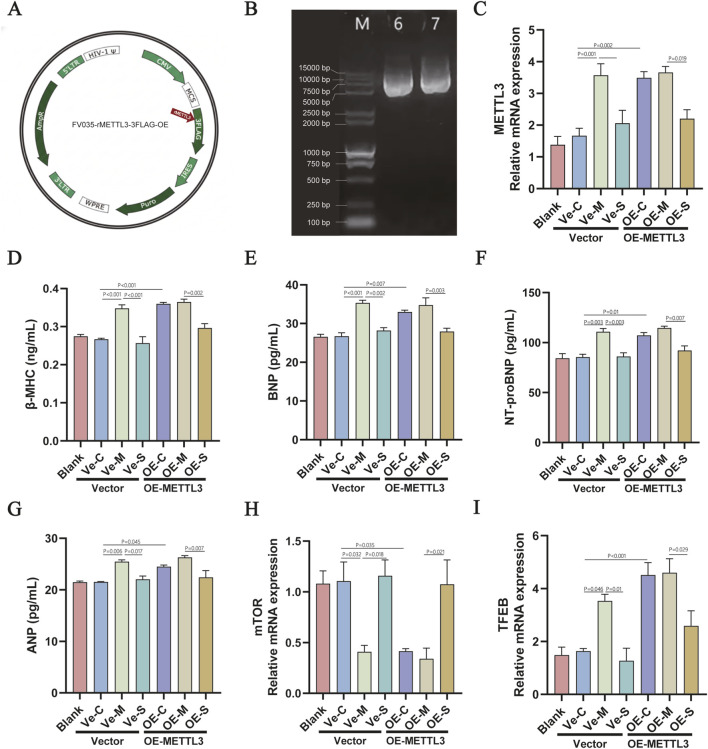
SYD improves H9c2 cell hypertrophy and autophagy pathway protein levels caused by overexpression of METTL3. **(A)** Schematic diagram of the recombinant lentiviral vector FV035-rMETTL3-3FLAG-OE. The rat Mettl3 gene was inserted into the MCS downstream of the CMV promoter and fused with a 3FLAG tag. **(B)** Identification of the recombinant plasmid by agarose gel electrophoresis. Lane M: DNA marker; Lane 6–7: Purified plasmid samples showing a single band at approximately 9.9 kb. **(C–E)** METTL3, mTOR, and TFEB mRNA levels of H9c2 cells in each group (n = 5). Cells were transduced with packaged lentivirus at a titer of 5 × 10^8^ TU/mL. **(F–I)** The content of hypertrophy markers (β-MHC, BNP, NT-proBNP, and ANP) in the supernatant of H9c2 cells in each group (n = 8). All column diagram data are shown as mean ± SEM. Ve-C: Negative vector control group; Ve-M: Negative vector model group; Ve-S: Negative vector SYD group; OE-C: Overexpression METTL3 control group; OE-M: Overexpression METTL3 model group; OE-S: Overexpression METTL3 SYD group.

To verify whether METTL3-mediated m6A modification affects the mTOR/TFEB autophagy pathway, we examined the MeRIP-seq results and found that the m6A methylation profiles of mTOR and TFEB were altered across different groups (Table 3). To further validate this mechanism, we constructed a lentiviral overexpression vector, FV035-rMETTL3-3FLAG-OE, to stably upregulate METTL3 expression in H9c2 cells. The integrity of the recombinant plasmid was confirmed by DNA sequencing and agarose gel electrophoresis, which showed a distinct band at approximately 9.9 kb. Following lentiviral packaging and transduction (titer: 5 × 10^8^ TU/mL), the successful upregulation of METTL3 was confirmed by RT-qPCR analysis (Figures 7A–C). Functional assays revealed that METTL3 overexpression exacerbated PE-induced cardiomyocyte hypertrophy, as evidenced by the significant elevation of hypertrophic markers, including β-MHC, BNP, NT-proBNP, and ANP (Figures 7D–G). Conversely, SYD treatment markedly attenuated these hypertrophic responses in both the vector-control and METTL3-overexpressing cells (Figures 7D–G). Furthermore, RT-qPCR analysis demonstrated that SYD reversed the METTL3-driven transcriptional suppression of mTOR and TFEB (Figures 7H,I). These findings establish METTL3 as a critical mediator of SYD’s effects on the mTOR/TFEB autophagy axis, delineating a novel epigenetic mechanism through which SYD modulates autophagic homeostasis in an m6A-dependent manner.

**TABLE 3:** Modification Sites of mTOR and TFEB.

Gene	Group	Methylation sites		Methylated RNA	Enrichment information	
		Chrom	Score	Peak_length	−10*log10 (pvalue)	fold_enrichment
mTOR	Control	chr5	20.19	201	20.19	5.37
		chr5	40.74	176	40.74	9.38
		chr5	30.15	201	30.15	8.09
		chr5	68.2	135	68.2	12.16
		chr5	78.61	249	78.61	13.71
		chr5	20.13	183	20.13	7.72
	Model	chr5	48.79	176	48.79	13.72
		chr5	30.95	249	30.95	9.15
	SYD	chr5	30.38	249	30.38	28.38
		chr5	52.56	183	52.56	39
		chr5	27.13	176	27.13	31.3
TFEB	Control	chr9	23.37	255	23.37	8.2
	Model	chr9	37.25	255	37.25	10.46
		chr9	31.12	255	31.12	6.71
	SYD	chr9	20.3	232	20.3	16.48

## 4 Discussion

This study aimed to investigate whether Shen-Yuan-Dan capsule (SYD) attenuates post-MI HF by regulating METTL3-mediated m6A methylation and mTOR/TFEB-dependent autophagy. Using integrated *in vivo* (MI mouse model) and *in vitro* (PE-induced cardiomyocyte hypertrophy) approaches, we demonstrated three key findings: First, SYD exerts cardioprotection by normalizing METTL3-driven m6A hypermethylation, reducing global m6A levels. Second, this epigenetic modulation directly impacts autophagic homeostasis, restoring physiological flux in ischemic myocardium while suppressing pathological overactivation in hypertrophic cardiomyocytes. Third, MeRIP-seq analysis identified mTOR and TFEB as m6A-modified targets, establishing a molecular link between SYD’s epitranscriptomic effects and autophagy regulation. And network pharmacology analysis suggests that SYD’s mechanism for treating HF may be related to autophagy pathways and could potentially target mTOR. These findings provide the first evidence that SYD ameliorates post-MI HF through METTL3/m6A-mTOR/TFEB signaling.

The inhibition of METTL3 by SYD aligns with recent evidence that METTL3 ablation mitigates cardiac remodeling by suppressing m6A-dependent pro-fibrotic gene expression (Gong et al., 2021). Our MeRIP-seq data extend these observations by identifying mTOR and TFEB—core autophagy regulators—as direct m6A targets. This parallels findings in ischemic heart injury, where METTL3 inhibition preserved TFEB activity, suggesting conserved RNA methylation mechanisms across ischemic pathologies (Son et al., 2019).

Our network pharmacology analysis reveals that its key ingredients, such as *Salvia miltiorrhiza*, *Corydalis*, and *Codonopsis pilosula*, may exert therapeutic effects on heart failure (HF) through the autophagy pathway, particularly by targeting mTOR. This finding aligns with existing literature that highlights the role of *Salvia miltiorrhiza* in protecting cardiovascular health and the involvement of autophagy regulators like mTOR in the pathophysiology of HF (Li et al., 2018). Additionally, compounds from *Corydalis* and *Codonopsis pilosula* have been shown to modulate autophagy and reduce myocardial damage, supporting our hypothesis that SYD’s therapeutic effects may be mediated through autophagy-related mechanisms (Kim et al., 2021; Sciarretta et al., 2018). The context-dependent autophagy modulation (activation *in vivo* vs. suppression *in vitro*) reflects SYD’s adaptive therapeutic effects. In post-MI hearts, SYD-enhanced autophagy likely clears damaged organelles, consistent with TFEB’s role in lysosomal biogenesis (Sciarretta et al., 2018). Conversely, in hypertrophy models, SYD inhibits mTOR-mediated excessive autophagy, mirroring the dual roles of autophagy in pressure-overloaded hearts (Cheng et al., 2017; Nakai et al., 2007). This divergence arises from distinct pathological demands: ischemia-triggered autophagy deficiency versus stress-induced autophagic overactivation (Eskelinen, 2019). The bidirectional nature of autophagy indicates that it can protect cells by clearing damaged structures under stress conditions, yet may also lead to autophagic cell death when excessively activated (Li M. et al., 2015). Thus, SYD facilitates the repair of damaged cells by enhancing autophagy *in vivo*, while simultaneously protecting myocardial cells by inhibiting excessive autophagy *in vitro*. This distinction reflects SYD’s adaptable regulatory capability over autophagic levels in diverse pathological conditions. The differential regulation of autophagy in these contexts suggests that SYD may have the potential for bidirectional modulation, promoting autophagy in ischemic heart disease while inhibiting it in non-ischemic hypertrophic pathology. This adaptable regulatory mechanism may offer insights for the future development of personalized treatment strategies, particularly concerning the adjustment of SYD dosage and administration based on patients' pathological types. However, we must acknowledge a potential discrepancy between the experimental preparation of SYD and the *in silico* components used for network pharmacology analysis. While our experiments utilized a traditional water decoction representing a complex mixture of constituents, the network pharmacology relied on database-derived metabolites and predicted targets (Duan et al., 2021). We recognize that the chemical profile of a laboratory-prepared extract may not perfectly mirror standard compound libraries in digital databases. Nevertheless, the high degree of consistency between our MeRIP-seq results and the network-predicted pathways—particularly the focus on the mTOR/autophagy axis—suggests that the major bioactive metabolites within the SYD decoction are likely well-represented in our computational model. Future research will focus on detailed phytochemical profiling, such as LC-MS/MS analysis, to definitively bridge the gap between *in silico* predictions and the actual chemical composition of the decoction.

SVST was selected as the positive control drug, which belongs to the class of Angiotensin Receptor Neprilysin Inhibitor (ARNI) drugs. It can improve the incidence and mortality of HF (Jhund and McMurray, 2016). SYD’s pharmacological effects in reducing cardiac dysfunction and remodeling parallels SVST, as evidenced by improved EF/FS values and suppressed serum biomarkers (BNP, β-MHC, TGF-β1). Crucially, SYD’s targeting of METTL3 addresses a critical gap in current HF therapies, offering a novel complementary strategy.

In this study, PE was employed to induce cardiomyocyte hypertrophy *in vitro* rather than Oxygen-Glucose Deprivation (OGD). While OGD effectively simulates acute ischemic injury, PE-induced hypertrophy better mimics the pathological signaling and maladaptive remodeling characteristic of the chronic ‘heart failure' phase following myocardial infarction (Lu et al., 2012). Since our primary objective was to investigate the therapeutic effects of SYD on post-MI cardiac dysfunction and autophagy homeostasis during the remodeling process, the PE model provided a more stable and relevant platform for assessing hypertrophic biomarkers and epigenetic regulation.

SYD alleviates post-MI HF through METTL3/m6A-dependent regulation of mTOR/TFEB-autophagy signaling, demonstrating adaptive modulation of autophagy across pathological contexts. These findings position RNA epitranscriptomics as a novel therapeutic axis for HF, bridging traditional medicine with cardiology.

This study has limitations. First, while H9c2 cells provide mechanistic insights, human iPSC-derived cardiomyocytes may better predict clinical relevance. Second, the specific SYD components mediating METTL3 inhibition remain uncharacterized; phytochemical studies should isolate active compounds (e.g., astragaloside IV or salvianolic acids). Third, there is a lack of dynamic observation of the autophagic flux and m6A methylation. Furthermore, we acknowledge that while our endpoint observations at 6 weeks *in vivo* and 24 h *in vitro* provide evidence of SYD’s therapeutic outcomes, the lack of real-time autophagic flux monitoring across multiple time points represents a limitation of this study. Additionally, although we confirmed SYD’s regulatory effects via METTL3-overexpression lentiviral vectors, the absence of standard pharmacological activators or inhibitors (e.g., Rapamycin or Chloroquine) as positive controls means that the precise, dynamic transitions of autophagic flux warrant further investigation in future studies. Finally, long-term SYD effects on cardiac electrophysiology warrant evaluation to ensure clinical safety.

## 5 Conclusion

In conclusion, the present study reveals that the traditional Chinese medicine compound SYD exerts a preventive and therapeutic effect against post-MI HF. Mechanistically, SYD inhibits METTL3, which in turn leads to a decrease in the m6A methylation level within cardiomyocytes. This reduction in m6A methylation further impacts the mTOR/TFEB - mediated autophagic homeostasis, thereby contributing to its cardioprotective action.

## Data Availability

The data presented in the study are deposited in the NCBI GEO repository, accession number GSE320263.
