# Supplementary material for: Shen-yuan-dan capsule inhibits METTL3-mediated m6A methylation to restore autophagy homeostasis and attenuate post-myocardial infarction heart failure
Source: Front Pharmacol. 2026 Mar 10;17:1661745. doi: 10.3389/fphar.2026.1661745 (PMC13008928; doi:10.3389/fphar.2026.1661745)
Supplement: Supplementary file 1 [file Supplementaryfile1.docx]

**
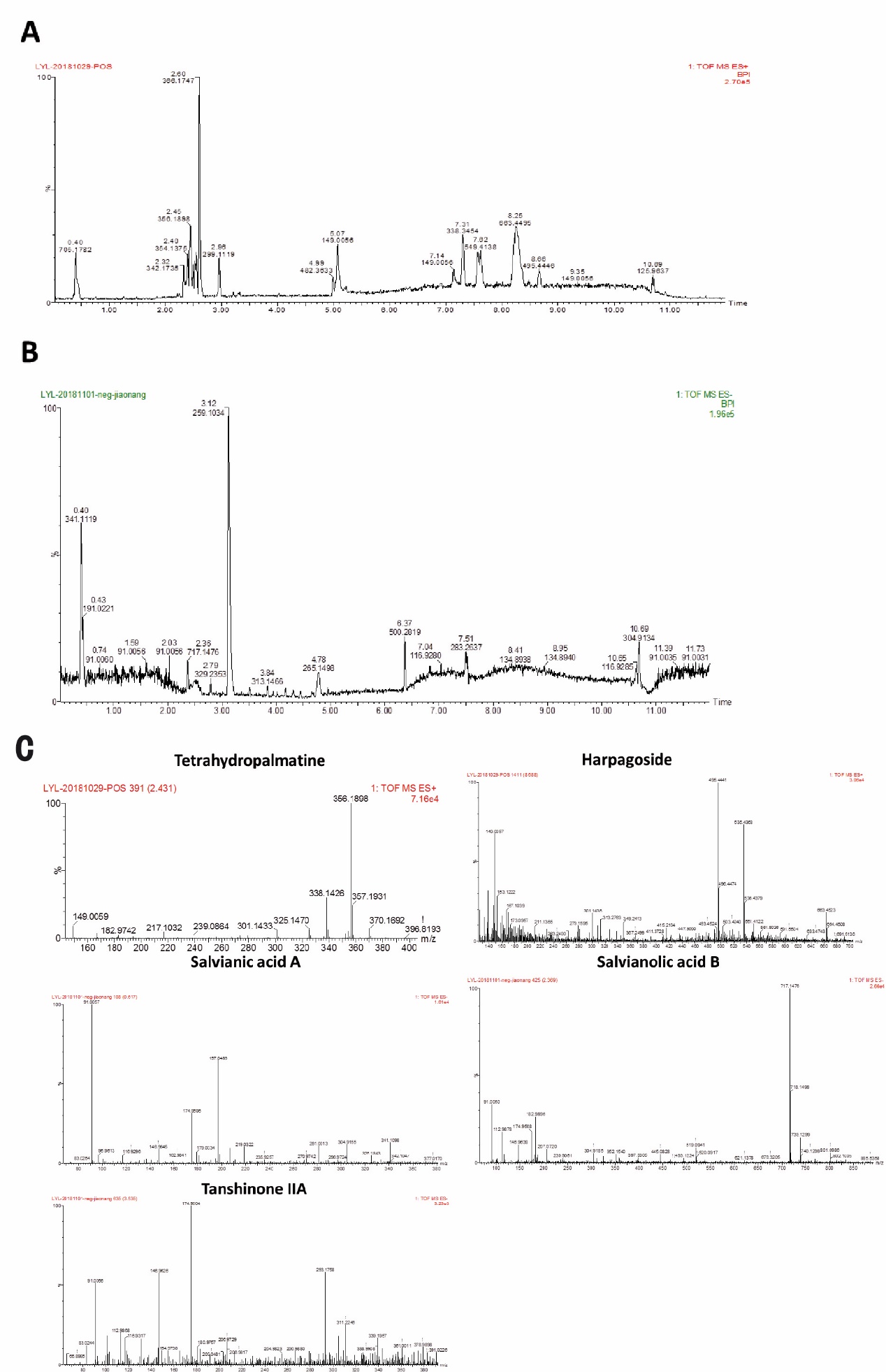
** Supplementary Figure S1. Identification of major components of SYDC. (A-B) The sample of SYDC was examined using UPLC–MS/MS. Data were collected and proceeded by software Masslynx 4.1. The positive (A) and negative (B) ion chromatograms of SYDC were shown as indicated. (C) The mass spectrograms of the main ingredients of metabolites SYDC.
